# Nonmedical use of prescription drugs in the European Union

**DOI:** 10.1186/s12888-016-0909-3

**Published:** 2016-08-04

**Authors:** Scott P. Novak, Anders Håkansson, Jose Martinez-Raga, Jens Reimer, Karol Krotki, Sajan Varughese

**Affiliations:** 1 Behavioral Epidemiology, RTI International, 3040 East Cornwallis Road, Research Triangle Park, NC 27709 USA; 2 Division of Psychiatry, Lund University, Malmö, Sweden; 3 University of Valencia, Hospital Universitario, Valencia, Spain; 4 Centre for Interdisciplinary Addiction Research, University Medical Centre Hamburg-Eppendorf Martinistrasse, Hamburg, Germany; 5 Statistical Sciences, RTI International, Washington DC, USA; 6 Shire, Lexington, MA USA; 7 Lund University, Faculty of Medicine, Department of Clinical Sciences Lund, Psychiatry, Lund, Sweden

## Abstract

**Background:**

Nonmedical prescription drug use (NMPDU) refers to the self-treatment of a medical condition using medication without a prescriber’s authorization as well as use to achieve euphoric states. This article reports data from a cross-national investigation of NMPDU in five European Countries, with the aim to understand the prevalence and characteristics of those engaging in NMPDU across the EU.

**Methods:**

A parallel series of self-administered, cross-sectional, general population surveys were conducted in 2014. Data were collected using multi-stage quota sampling and then weighted using General Exponential Model. A total of 22,070 non-institutionalized participants, aged 12 to 49 years, in 5 countries: Denmark, Germany, Great Britain, Spain, and Sweden. Lifetime and past-year nonmedical use of prescription medications such as stimulants, opioids, and sedatives were ascertained via a modified version of the World Health Organization’s Composite International Diagnostic Interview. Information about how the medications were acquired for NMPDU were also collected from the respondent.

**Results:**

Lifetime and past-year prevalence of nonmedical prescription drug use was estimated for opioids (13.5 and 5.0 %), sedatives (10.9 and 5.8 %), and stimulants (7.0 and 2.8 %). Germany exhibited the lowest levels of NMPDU, with Great Britain, Spain, and Sweden having the highest levels. Mental and sexual health risk factors were associated with an increased likelihood of past-year nonmedical prescription drug use. Among past-year users, about 32, 28, and 52 % of opioid, sedative, and stimulant nonmedical users, respectively, also consumed illicit drugs. Social sources (sharing by friends/family) were the most commonly endorsed methods of acquisition, ranging from 44 % (opioids) to 62 % (sedatives). Of interest is that Internet pharmacies were a common source of medications for opioids (4.1 %), stimulants (7.6 %), and sedatives (2.7 %).

**Conclusions:**

Nonmedical prescription drug use was reported across the five EU countries we studied, with opioids and sedatives being the most prevalent classes of prescription psychotherapeutics. International collaborations are needed for continued monitoring and intervention efforts to target population subgroups at greatest risk for NMDU.

## Background

Nonmedical prescription drug use (NMPDU) typically encompasses consumption of a medication that is not prescribed to a user or that is consumed in a manner not intended by the prescriber (e.g. taking higher doses, using non-approved routes of administration). It also captures situations in which the medication is obtained illegally (e.g. purchased through a dealer or the Internet) or under false pretenses (e.g. doctor shopping or feigning symptoms). In addition to the source of acquisition and the route of administration, the underlying motivations for use may include using the medication for self-treatment of a physical and/or mental health problem or for tension reduction/relaxation/euphoria [1]. NMPDU is among the leading public health issues in the United States (US) [2–5]. Prescriptions written for central nervous system (CNS) medications in the US has dramatically increased over the past decade, primarily in the therapeutic classes involving opioids, sedatives, and stimulants [6, 7]. Inspection of the number of peer-reviewed publications and media reports focusing on prescription drug abuse, misuse, and diversion over the past several years suggests that NMPDU is more widespread in the US than other nations, including the European Union (EU). This lack of attention is surprising, as the EU has also witnessed a rise in the number of prescriptions across these same therapeutic classes [8]. In tandem, there has also been an increase in emergency room visits and drug treatment admissions for prescription CNS medications in the EU [9–12]. Yet, the EU lacks a systematic method for identifying and monitoring trends in NMPDU over time, so its prevalence and associated user characteristics are largely unknown.

The US has arguably the most sophisticated and diverse drug abuse surveillance systems in the world, ranging from direct estimates of self-reported drug use to administrative data on drug arrests, seizures, and drug treatment admissions [13–20]. In the EU, the European Monitoring Centre for Drugs and Drug Addiction (EMCDDA) is the agency charged with coordinating information about specific drugs, and their health and social consequences. The agency was established in 1993, and disseminates reports using data provided from its member nations. Most of its data are from secondary sources, such as arrests, seizures, and drug treatment admissions [21]. While the US has numerous opportunities for funding epidemiological studies on NMPDU, the EU has comparatively fewer sources of funding to support new data collection efforts. On occasion, the EMCDDA is able to acquire primary survey data from dedicated government funding.

Due to the lack of a comprehensive surveillance system and funding for NMPDU studies in the EU, most of what is known comes from studies conducted in the US. A consistent finding in the literature from the US and the EU is that NMPDUs often engage in polydrug use [22, 23]. However, there is also a group of persons who engage in NMPDU but who do not engage in poly-drug use [24–26]. Of importance is that few studies have specifically examined these two groups, including providing estimates of the number of users relative to the total group of persons engaging in NMPDU, as well as their defining characteristics.

NMPDU has received a considerable amount of attention in the US, but anecdotal evidence suggests that other nations are experiencing dramatic increases as well [27]. Drug abuse is becoming more global in nature. With regard to prescription medications, global studies are critical to pharmacovigilance, which is the process of monitoring the individual and population-level health effects after medications have been approved for retail sales. This process has relevance for numerous stakeholders, as NMPDU is a key metric that provides a measure of a medication’s abuse liability and safety profile for clinicians, payers, and policy-makers. Prescription drug abuse is a prominent topic in many Internet chat-rooms, including information about availability, consumption, and side-effects. These discussions are international in focus, as participants come from many different types of nationalities. The international borders that separate the drug trade in each country are also eroding. For example, drug users are even able to make anonymous purchases through online pharmacies [28–30]. There is need to understand whether the high levels of NMPDU observed in the United States are similar in other EU countries. The objectives of this study are to examine the prevalence NMPDU in the EU, including a set of analyses to identify the subgroups at greatest risk and the methods of acquisition.

## Methods

### Study population and design

The European Union Medicine Study (EU-Meds Study) was a parallel series of national surveys conducted in Denmark, Germany, Great Britain, Spain, and Sweden. The target sample age range was 12 to 49 years, which is the typical period of initiation for substance use around the globe. The study design used a quota sampling methodology [31]. First, targets were identified in each country based on sex, age, and marital status. Quotas were also set based on characteristics that were highly associated with NMPDU, such as tobacco and marijuana use [32]. The overall recruitment targets were set to achieve an initial sample proportionate to size in each country. The targets were monitored each week, and once a quota was met, no additional persons matching those characteristics were eligible for the study. The second stage, described later, adjusted the sample using weighting under the General Exponential Model (GEM) weighting [33]. This step further calibrated the quota sample much in the same way a post-hoc weight is applied to a probability sample. Both methods are used to achieve the same goal: a nationally representative sample. A brief note on the measurement of race in this study. Typically, EU countries do not measure race in the same way, so we limited our investigation to the measure of race based on white and non-white. We compared our racial characteristics to available data and it appears that the distribution of non-whites in the EU countries we studied ranges between 87 % (Great Britain) to Denmark (98 %).

Participants were recruited through a diverse range of methods, first starting with advertisements in local newspapers, then moving to street-intercept recruitments in open-air drug markets, needle exchanges, homeless shelters, and parks/libraries. Another method of recruitment was to use market research lists to recruit either adults or adults with children. Because of ethical requirements in the protection of minors in the EU, the study sample and recruitment/data collection procedures were divided into two age groups: one for youth (ages 12 to 17) and another for adults (ages 18 to 49). The youth sample was required to complete the survey of one of the onsite research sites. The facilities were located in geographically centralized areas to ensure a diverse range of participants across the rural/suburban/urban environments. Once recruited, the youths were asked to arrive on-site with their legal guardian. Adults were provided with a unique web link to take the survey on their own. Identity was verified using a unique passcode for each respondent. Individuals were paid approximately €50 Euros. A total of 22,075 individuals completed the survey, but 5 respondents were eliminated due to significant missing data (>0 %) on the survey items. The final sample was 22,070, which included 2,032 youths and 20,038 adults. The consent rate for the overall study was 78 % based on the number of individuals who screened eligible and those who consented to be part of the study.

### Questionnaire and measures

The participants were able to take the survey in English and also in each country’s native language (i.e. Danish, German, Swedish, and Spanish). The measures were all based on self-report, including demographics such as age, sex, and race. The surveys were initially developed using the World Health Organization’s Composite Diagnostic Interview Schedule (CIDI) [34]. CIDI has been adapted for self-administration as part of a diagnostic calibration [35]. The primary outcome measures were based on the NMPDU for three of the most commonly abused therapeutic classes: stimulants, opioids, and sedatives (e.g., tranquilizers, benzodiazepines). A respondent was first provided with a written introduction that defined nonmedical use as either (a) self-treatment using a prescription that was not their own or (b) misuse of the product for euphoria. For instance, the questions starts outs with an explanation that…”we are not interested in your use of over-the-counter medications that you cannot obtain without a doctor’s or pharmacist’s permission.” Next, the question asks the respondent whether they have ever used medication “for euphoria, to obtain a high” and or “to self-treat a medical problem using mediation that was not specifically prescribed for you.”

The primary independent variables were ascertained by self-report measures based on whether the respondent had endorsed that they were prescribed stimulants, opioids, or sedatives for any reason by a licensed prescriber. A single question asked if the respondent was diagnosed with Attention Deficit Disorder (with or without Hyperactivity) and another question ascertained a diagnosis of HIV/AIDS. An additional question asked if the respondent was ever arrested for any legal reason, regardless of whether they were incarcerated as a result of the arrest. Finally, serious psychological distress was measured by the Kessler Six Item (K6) measure, which included items on depressive mood, self-derogation, anxiety, and role impairment. The K6 has been validated as a screening measure for mood and anxiety disorders.

### Analysis

A goal of the sampling design was to produce population-based estimates within each country. The pool of respondents was selected from a quota sample methodology; within this design, the selection probabilities are unknowable because the sample is based on multiple recruitment channels rather than a single sampling frame. After the data collection was completed in each country, the study statistician reviewed the bias in the final sample and the targets. The first step in the analysis plan was to create weights for respondents in each country, generalizable to the larger population aged 12 to 49. This step is similar to post-hoc weight adjustments that are typically used in probability-based samples. The method of calibration weighting was applied to the raw survey data to make the inferences generalizable to the larger population of non-institutionalized residents in each country. In order to calculate the post-stratification weights, the sample was first divided into two separate population groups: youth (ages 12 to 17) and adults (ages 18 to 49). The available data varied by country, but the main variables for which distributions were available included age, sex, marital status, employment status, education, and nativity. Two additional substance use variables were included: alcohol and cigarettes were also included. The final weighted data closely approximated the final demographic distributions in each country as well as substance use characteristics associated with the primary outcomes of interest. Additional sensitivity analyses (not shown) compared the final weighted results to published data. Once the sample weights were finalized, the analyses were conducted on the weighted data. Cross tabulations and multivariable logistic regression methods were used via SUDAAN (release 11.1, RTI International). For the logistic regression models, separate analyses were estimated for each therapeutic class. Because it is possible that NMPDUs may engage in use for therapeutic reasons as well as euphoria, we examined the association comparing those who just engaged in NMPDU relative to those engaging in NMPDU and illicit drug use. Put differently, it is possible that initial NMPDU may serve as a gateway to other substances by exposing users to the physiological and socio-cultural aspects of substance use. There is also a possibility of a selection effect, whereby persons using illicit drugs are at higher risk for NMPDU. With cross-sectional data, we are unable to resolve this question; yet, data from this study provide an examination of the preliminary associations that can be investigated in future studies that contain developmentally sensitive measures collected on the same set of individuals over time. Such information will help identify the factors that differentiate NMPDU from non-users, and then between NMPDUs and poly-drug use involving illicit drug use. This outcome was coded as a binary variable (0 = no NMDPU and 1 = any NMPDU) for the first analysis, and then as a binary variable (0 = NMDPDU only and 1 = NMPDU and ILLICIT DRUGS), except that the analyses are subset to those with any NMPDU.

## Results

### Prevalence of nonmedical prescription drug use

The demographics are provided in Table 1 but they are representative to the demographics in each of the 5 EU countries, including race, gender, and age. The overall lifetime and past-year prevalence estimates of NMPDU in the combined sample (Table 1) indicated that opioids were the most commonly endorsed medication for the overall lifetime use (13.5 %) whereas sedatives were the most commonly endorsed medication for the overall past-year use (5.8 %). Stimulants were the least commonly endorsed medication for both lifetime (7 %) and past-year (2.8 %) use. Illicit drug use was 38.1 % for lifetime use and 11.7 % for use in the past-year. Marijuana was the most frequently used illicit drug in both the lifetime and past-year estimates.

**Table 1:** Characteristics of EU-Meds Study, 2014

Characteristic	Percentage^a^	Total No.^b^
Country		
Denmark	12.4	2,732
Germany	24.9	5,511
Great Britain	25.3	5,572
Spain	24.9	5,507
Sweden	12.5	2,748
Sex		
Male	50.3	9,725
Female	49.7	12,245
Race		
White	93.0	17,475
Non-white	7.0	1,413
Age, years		
12–17	9.2	2,032
18–29	32.9	7,048
30–49	57.9	12,990
Marital Status^c^		
Never married	45.5	8,775
Married/cohabitating	33.2	7,032
Divorced/separated/widowed	21.3	4,231
Employment		
Full/Part time	52.5	12,133
Unemployed	11.2	2,268
Student	18.4	3,950
Not in labor force	17.9	3,719
Lifetime NMPDU		
Stimulant	7.0	1,302
Opioid	13.5	2,682
Sedative	10.9	2,203
Past-Year NMPDU		
Stimulant	2.8	498
Opioid	5.0	949
Sedative	5.8	1,099
Illicit Drug Use, Lifetime^d^	38.1	7,856
Illicit Drug Use, Past-Year^d^	11.7	2,200

*NMPDU* nonmedical prescription drug use

^a^Estimate based on weighted data

^b^Sample size is unweighted

^c^Sample restricted to ages 18 or older

^d^Illicit drug use includes marijuana, cocaine, heroin, methamphetamine, hallucinogens, inhalants, and designer drugs

### Characteristics of nonmedical prescription drug users

The prevalence of NMPDU varied across numerous subgroups, as shown in Table 2. Great Britain had by far the highest nonmedical use of prescription stimulants for both past-year (3.9 %) and lifetime (9.1 %) estimates. The remaining four countries were closely aligned in past-year stimulant use, ranging from 2.2 % (Germany) to 2.6 % (Sweden). This grouping was similar for lifetime estimates, with the prevalence estimates ranging from 5.8 % (Germany) to 6.8 % (Spain). Nonmedical stimulant use was the least common in Germany for both past-year (2.2 %) and lifetime (5.8 %) estimates.

**Table 2:** Lifetime and past-year prevalence of nonmedical prescription drug use in selected subgroups, EU-Meds, 2014

	Past-Year, %^a^ (SE)^b^			Lifetime, %^a^ (SE)^b^		
Characteristic	Stimulants	Opioids	Sedatives	Stimulants	Opioids	Sedatives
Country						
Denmark	2.5 (0.4)	4.4 (0.5)	3.8 (0.5)	6.0 (0.6)	11.6 (0.8)	7.8 (0.6)
Germany	2.2 (0.2)	2.9 (0.2)	2.8 (0.2)	5.8 (0.3)	9.6 (0.4)	5.5 (0.3)
Great Britain	3.9 (0.5)	6.2 (0.5)	5.7 (0.5)	9.1 (0.6)	14.6 (0.7)	10.1 (0.6)
Spain	2.4 (0.3)	6.8 (0.5)	9.2 (0.6)	6.8 (0.4)	18.3 (0.7)	17.9 (0.7)
Sweden	2.6 (0.4)	3.8 (0.5)	7.5 (0.6)	6.1 (0.6)	11.3 (0.7)	12.4 (0.8)
*P*-Value	*P* < .001	*P* < .001	*P* < .001	*P* < .001	*P* < .001	*P* < .001
Sex						
Male	3.8 (0.3)	5.7 (0.3)	6.4 (0.4)	9.5 (0.4)	15.4 (0.5)	11.6 (0.4)
Female	1.8 (0.2)	4.2 (0.2)	5.2 (0.3)	4.4 (0.2)	11.6 (0.4)	10.2 (0.3)
*P*-Value	*P* < .001	*P* < .001	*P* < .001	*p* < .001	*P* < .001	*P* = .016
Race						
White	2.5 (0.2)	4.9 (0.2)	5.4 (0.2)	6.5 (0.3)	13.5 (0.4)	10.6 (0.3)
Non-white	6.0 (1.0)	8.0 (1.1)	9.0 (1.2)	14 (1.3)	19.5 (1.5)	13.7 (1.3)
*P*-Value	*P* < .001	*P* < .001	*P* < .001	*P* < .001	*P* < .001	*P* < .015
Age, years						
12–17	1.0 (0.2)	1.6 (0.3)	1.2 (0.2)	1.9 (0.3)	3.5 (0.4)	1.6 (0.3)
18–29	3.6 (0.3)	5.1 (0.4)	6.3 (0.4)	8.9 (0.5)	13.1 (0.6)	10.2 (0.5)
30–49	2.6 (0.2)	5.5 (0.3)	6.3 (0.3)	6.6 (0.3)	15.3 (0.4)	12.7 (0.3)
*P*-Value	*P* < .001	*P* < .001	*P* < .001	*P* < .001	*P* < .001	*P* < .001
Marital Status^c^						
Never married	2.9 (0.2)	4.6 (0.2)	5.8 (0.3)	6.8 (0.3)	11.9 (0.4)	10.5 (0.4)
Married/cohabitating	3.2 (0.3)	6.1 (0.4)	5.9 (0.3)	7.7 (0.5)	16.4 (0.6)	11.7 (0.5)
Divorced/separated/widowed	1.9 (0.3)	4.3 (0.4)	5.7 (0.5)	6.2 (0.5)	13.0 (0.6)	10.6 (0.6)
*P*-Value	*P* = .027	*P* = .002	*P* = .908	*P* = .074	*P* < .001	*P* = .100
Employment						
Full/Part time	2.9 (0.3)	4.9 (0.3)	5.4 (0.3)	7.4 (0.3)	14.2 (0.4)	10.8 (0.4)
Unemployed	3.3 (0.5)	6.4 (0.7)	8.3 (0.7)	8.5 (0.8)	17.6 (1.1)	16.1 (1.1)
Student	1.8 (0.3)	2.6 (0.3)	3.9 (0.5)	4.3 (0.4)	7.1 (0.6)	5.6 (0.5)
Not in labor force	2.9 (0.4)	7.1 (0.6)	7.7 (0.6)	7.4 (0.6)	15.4 (0.8)	13.3 (0.8)
*P*-Value	*P* = .022	*P* < .001	*P* < .001	*P* < .001	*P* < .001	*P* < .001

^a^Estimate based on weighted data. ^b^Sample size is unweighted. ^c^Sample restricted to ages 18 or older. *P*-Value tests (Wald) for differences in NMPDU between levels of characteristic

SE, standard error

The highest prevalences of nonmedical opioid use were in Spain (6.8 % past-year, 18.3 % lifetime) and Great Britain (6.2 % past-year, 14.6 % lifetime). Germany had the lowest estimates for opioid nonmedical use. Spain and Sweden had the most prevalent use of sedatives, followed by Great Britain and Denmark. Germany had the lowest percentage of users in the population for past-year (2.8 % for stimulants) and lifetime (5.5 % for sedatives) NMPDU.

The lifetime and past-year prevalence estimates of the three therapeutic classes of prescription CNS medications indicated that usage was more common among males relative to females, non-whites relative to whites, and those who were unemployed compared with other levels of employment. Those aged 12 to 17 years were also at lower risk of NMPDU compared with those aged 18 years or older at the time of survey administration.

In addition to the bivariable comparisons, we also examined the association between NMPDU and selected health characteristics and past-year NMPDU. As shown in Table 3, having received a prescription for the particular outcome drug was associated with an elevated risk of nonmedical use. For example, having a prescription for a pain reliever was associated with nearly an eight times higher risk of nonmedical use of prescription pain relievers. The risk was ten times higher for sedatives and seven times higher for stimulants. Other mental health-related conditions were also powerful correlates of NMPDU, including non-specific psychological distress and attention-deficit/hyperactivity disorder (ADHD). Early childhood risks in terms of being arrested prior to age 15, and current sexually transmitted disease (STD) and human immunodeficiency virus (HIV) status were also associated with higher odds of past-year use of all three types of prescription medications.

**Table 3:** Predictors of past-year nonmedical prescription drug abuse, EU-Meds, 2014

	Stimulants			Opioids			Sedatives		
	2.8 %^a^ (0.4)^b^ N = 22,070			5.0 %^a^ (0.5)^b^ N = 22,070			5.8 %^a^ (0.7)^b^ N = 22,070		
	O.R.	95 % C.I.	*P*-Value	O.R.	95 % C.I.	*P*-Value	O.R.	95 % C.I.	*P*-Value
Country									
Great Britain		1.0			1.0			1.0	
Denmark	0.6	0.4–0.9	.001	0.7	0.5–0.9	.015	0.7	0.5–0.9	.006
Germany	0.5	0.4–0.7	<.001	0.5	0.4–0.6	<.001	0.5	0.4–0.7	<.001
Spain	0.6	0.4–0.8	.003	1.1	0.9–1.4	.383	1.7	1.3–2.1	<.001
Sweden	0.7	0.5–0.9	.032	0.6	0.4–0.8	.001	1.3	1.0–1.7	.029
Wald Chi-Square (DF)-P	Chi = 4.2, 4df, *P* = .002			Chi = 19.3, 4df, *P* < .001			Chi = 39.3, 4df, *P* < .001		
Sex									
Male		1.0			1.0			1.0	
Female	0.5	0.3–0.8	.002	0.7	0.6–0.9	<.000	0.8	0.7–0.9	.008
Age, years									
12–17		1.0			1.0			1.0	
18–29	3.6	2.3–5.7	<.001	3.4	2.3–4.9	<.001	5.5	3.5–8.2	<.001
30–49	2.5	1.6–4.0	<.001	3.6	2.5–5.3	<.001	5.4	3.6–8.2	<.001
Wald Chi-Square (DF)-P	Chi = 15.5, 2df, *P* = <.001			Chi = 24.0, 2df, *P* = <.001			Chi = 33.7, 2df, *P* = <.001		
Prescribed (outcome drug)^c^									
No		1.0			1.0			1.0	
Yes	7.8	6.1–10.2	<.001	8.8	7.3–10.6	<.001	10.5	8.6–12.6	<.001
Serious Psych Distress									
No		1.0			1.0			1.0	
Yes	4.5	3.5–5.8	<.001	3.2	2.6–3.9	<.001	4.2	3.5–5.0	<.001
ADHD Dx									
No		1.0			1.0			1.0	
Yes	9.5	7.2–12.5	<.001	3.5	2.6–4.6	<.001	5.1	3.9–6.5	<.001
Sexually Transmitted Disease									
No		1.0			1.0			1.0	
Yes	7.2	4.8–10.9	<.001	4.6	3.1–6.9	<.001	3.9	2.7–5.6	<.001
HIV									
No		1.0			1.0			1.0	
Yes	15.1	7.7–29.3	<.001	18.9	10–34	<.001	12.2	6.5–22.7	<.001
Arrested < Age 15									
No		1.0			1.0			1.0	
Yes	2.6	2.1–3.5	<.001	2.9	1.9–2.7	<.001	2.1	1.8–2.5	<.001

Reference level O.R. = 1.0. All estimates adjusted for complex sampling design using SUDAAN (release 10.1). Wald Test used for all *P*-Values

*Dx* diagnosis, *HIV* human immunodeficiency virus, *O.R.* odds ratio, *C.I.* confidence interval

^a^Weighted Percentage

^b^Weighted Standard Error. Ever prescribed refers to whether the respondent was ever prescribed the outcome drug (Stimulants, Opioids, or Sedatives. Illicit drug use includes any of the following: marijuana, cocaine, heroin, inhalants, designer drugs

### Polydrug use involving nonmedical prescription drug use and illicit drug use

As illicit drug use is a well-documented risk factor for NMPDU and may also be a consequence of initiation, the next set of analyses (Table 4) sought to examine differences between two subgroups of users: those who use only prescription medications and those who engage in poly-drug use involving illicit drugs. There were country-wide differences only for opioids and sedatives, with Great Britain being the country with the highest rate of poly-drug use. Among past-year nonmedical sedative users, 48 % of past-year users in Great Britain also used illicit drugs, compared to 26 % in Germany, 22 % in Denmark and Sweden, and 20 % in Spain. Among past-year nonmedical prescription opioid users, 43 % of past-year users in Great Britain also used illicit drugs, compared to 41 % in Sweden, 30 % in Germany, 24 % in Denmark, and 21 % in Spain.

**Table 4:** Predictors of past-year co-occurring nonmedical prescription drug abuse and co-occurring illicit drug use, EU-Meds, 2014

	Stimulants			Opioids			Sedatives		
	52.5 %^a^ (0.5)^b^ *N* = 498			32.1 %^a^ (0.4)^b^ *N* = 949			28.3 %^a^ (0.3)^b^ *N* = 1,099		
	O.R.	95 % C.I.	*P*-Value	O.R.	95 % C.I.	*P*-Value	O.R.	95 % C.I.	*P*-Value
Country									
Great Britain	1.0			1.0			1.0		
Denmark	0.9	0.4–1.9	.765	0.4	0.2–0.8	.006	0.3	0.1–0.6	<.001
Germany	0.5	0.3–0.9	.018	0.6	0.3–0.9	.025	0.4	0.2–0.7	<.001
Spain	0.6	0.3–1.1	.065	0.4	0.2–0.6	<.000	0.3	0.2–0.4	<.001
Sweden	0.8	0.4–1.7	.529	0.9	0.5–1.7	.744	0.3	0.2–0.5	<.001
Wald Chi-Square (DF)-P	Chi = 1.8, 4df, *P* = .111			Chi = 5.4, 4df, *P* < .001			Chi = 7.8, 4df, *P* < .001		
Sex									
Male	1.0			1.0			1.0		
Female	0.5	0.3–0.7	.002	0.6	0.4–0.9	.013	0.6	0.4–0.9	.007
Age, years									
12–17	1.0			1.0			1.0		
18–29	0.9	0.3–2.2	.748	1.2	0.5–2.6	.655	0.9	0.4–2.1	.764
30–49	0.9	0.3–2.0	.628	0.8	0.4–1.7	.494	0.6	0.3–1.4	.256
Wald Chi-Square (DF)-P	Chi = 0.87, 2df, *P* = .871			Chi = 2.5, 2df, *P* = .083			Chi = 2.0, 2df, *P* = .133		
Prescribed [outcome drug]^c^									
No	1.0			1.0			1.0		
Yes	0.7	0.4–1.2	.173	0.9	0.6–1.4	.837	0.8	0.5–1.3	.354
Serious Psych Distress									
No	1.0			1.0			1.0		
Yes	1.8	1.1–2.9	.015	2.2	1.5–3.3	<.001	1.8	1.3–2.7	<.001
ADHD Dx									
No	1.0			1.0			1.0		
Yes	1.0	0.6–1.7	.969	1.6	0.9–2.9	.091	1.4	0.8.2–2.2	.117
Sexually Transmitted Disease									
No	1.0			1.0			1.0		
Yes	2.4	1.2–4.8	.018	5.2	2.5–10.7	<.001	2.8	1.4–5.5	.003
HIV									
No	1.0			1.0			1.0		
Yes	0.9	0.3–2.6	.768	1.4	0.6–3.7	.456	1.8	0.7–4.8	.207
Arrested < Age 15									
No	1.0			1.0			1.0		
Yes	1.7	1.1–2.9	.023	2.3	1.5–3.4	<.001	1.9	1.3–2.8	.002
Source of NMPDU									
Social (Friend/Family)	1.0			1.0			1.0		
Dealer/Theft/Fake	1.9	1.2–2.9	.008	2.6	1.8–3.9	<.001	1.8	1.1–2.7	<.001
Non-oral Routes of Administration									
No	1.0			1.0			1.0		
Yes	2.1	1.3–3.4	<.001	1.3	0.9–2.0	.138	1.2	0.8–1.8	.478
Motivation for Use-Euphoria									
No	1.0			1.0			1.0		
Yes	1.9	1.2–3.1	.008	4.8	3.1–7.4	<.001	3.5	2.0–6.1	<.001

Reference level O.R. = 1.0; All estimates adjusted for complex sampling design using SUDAAN (release 10.1). Wald Test used for all *P*-Values

*Dx* diagnosis, *HIV* human immunodeficiency virus, *NMPDU* nonmedical prescription drug use, *O.R* odds ratio, *C.I* confidence interval

^a^Weighted percentages

^b^Weighted standard error. Note: outcome coded 0 = No Co-Occurring Illicit Drug Use, 1 = Any Co-Occurring Illicit Drug Use

^c^Ever prescribed refers to the respondent receiving a prescription for the outcome drug (Stimulants, Opioids, or Sedatives. Illicit drug use includes any of the following: marijuana, cocaine, heroin, inhalants, designer drugs

Females were about half as likely to engage in concomitant illicit drug use as males. Interestingly, there were no age-related differences across any of the three drug-classes investigated. Conversely, those with serious psychological distress were almost twice as likely to engage in illicit drug use with NMPDU. ADHD and HIV did not confer additional risk, yet those with an STD were more likely to have reported poly-drug use than those without STDs. Similarly, those with childhood arrests were more likely to engage in illicit drug use with NMPDU.

### Sources of access

Those who engaged in theft, forgery, or doctor shopping were about 2 to 2.5 times more likely to have also used illicit drugs. For example, past-year users of stimulants were about 90 % (odds ratio [O.R.] = 1.9, 95 % confidence interval [C.I.] = 1.2–2.9) more likely to also use illicit drugs if they reported non-social sources of access. Those using for euphoria or other non-treatment reasons were far more likely to have also consumed illicit drugs in the past year, with the risk being higher for opioids (O.R. = 4.8, 95 % C.I. = 3.1–7.4), sedatives (O.R. = 3.5, 95 % C.I. = 2.0–6.1), and stimulants (O.R. = 1.9, 95 % C.I. = 1.2–3.1).

**Fig. 1:**
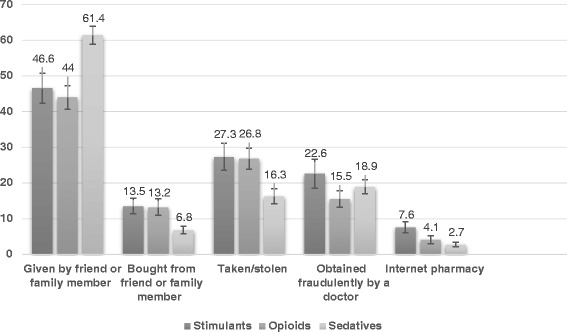
Sources of nonmedical prescription drug use among past-year users, EU-Meds, 2014

Figure 1 presents the prevalence estimates for different types of sources. Among the past-year users of stimulants, 71 % reported one source for obtaining the medication for nonmedical use, 16 % reported two sources, and 13 % reported three or more sources. This distribution was similar for opioids (one source = 73 %, two sources = 18 %, three or more sources = 9 %) and sedatives (one source = 80 %, two sources = 12 %, three or more sources = 8 %). Across all three classes of mediations, a friend or family member was the most common method of acquisition for stimulants (46.6 %), opioids (44 %), and sedatives (61.4 %). The next most common method of acquisition was those who reported taking them from another person without their knowledge. Of note, Internet purchases were the least common methods for stimulants (8 %), opioids (4 %), and sedatives (3 %).

## Discussion

This study documented the cross-national prevalence of NMPDU and associated characteristics of users in five EU countries. The results reported here provide important comparative estimates for cross-national investigations. For instance, data from the 2013 National Survey on Drug Use and Health (NSDUH), which is among the leading epidemiological sources of drug abuse in the United States, revealed that 20 % of those aged 12 years or older reported any lifetime use of a prescription psychotherapeutic [36]. The lifetime estimates for the EU-Meds Study ranged between 7 and 13 %. Despite methodological differences across studies, the findings suggest that the prevalence estimates in the EU are likely to be lower than the prevalence of nonmedical prescription drug use in the US. There were no data available on the health consequences of NMPD in this study, such as motor vehicle accidents or emergency department visits. Therefore, it is possible that the EU and the US may share similarities in the level of use, but the individual and societal consequences associated with use may differ. Compared to the EU, the US has a higher rate of motor vehicle accidents and unintentional overdoses that can be attributable to prescription medicines [2, 37–39]. The limited data from the EU suggest that much of the burden is attributable to illicit drugs rather than prescription-type medications [40]. Although Europeans may be using these medications on their own or without their physician’s knowledge, the public health consequences may not have reached levels similar to those in the US.

This epidemiological study also examined different patterns of exposure to prescription medications. One of the pathways to NMPDU is commonly referred to as “iatrogenic addiction” [24]. This pathway leads to nonmedical use after receiving treatment for a legitimate medical condition. Therefore, persons initiated into NMPDU through this pathway are less likely to have a history of poly-drug use. These data show that persons receiving a prescription had a higher likelihood of engaging in NMPDU than persons without a prescription. However, poly-drug users were not at differential risk than persons engaging only in NMPDU. A note of caution is that this study is population-based, and did not link exposure from a specific prescription to long-term outcomes. Instead, the survey asked about the receipt of medications used to treat common conditions, and the subsequent use of prescription medications nonmedically. Given the limitations of observational data, this finding should not be construed as a recommendation against prescribing medications to treat legitimate conditions. Any such recommendation should be based on methodologies that allow for a greater ability to address confounding than an observational design. Future studies are needed to more thoroughly investigate the role of patient access and the likelihood of NMPDU. In the EU, some prescription medications are available directly from a pharmacist. For example, codeine is available from a pharmacist in several EU countries. Additional studies are needed to examine how differences in prescribing are related to the onset and course of NMPDU.

This study also found that social sources (e.g. a family member or friend) were the most prevalent methods of acquisition, which is similar to findings in the US and other international studies [13, 41, 42]. However, the patterns we observe in the self-reported methods of acquisition regarding medications for NMPDU suggest that the Internet is becoming less common for accessing prescription medications for NMPDU in the US [43]. Inciardi and colleagues conducted an analysis of 2006 NSDUH data, showing that among those using the Internet to acquire medications, stimulants were the most common prescription drug purchased on the Internet (4 %), followed by sedatives (1 %), and opioids (0.5 %) [30]. According to a 2009 study conducted by the Pew Research Center, approximately 74 % of adults aged 18 years or older in the US use the Internet [44]. Compared with the US, preliminary evidence indicates that a larger proportion of citizens in the EU use the Internet, and also use it more frequently [45]. The US has been aggressive in regulating Internet pharmacies over the past several years, so it is unclear whether differences in the number of Internet users or aggressive policies may explain any between-country differences between the EU and other countries [46, 47].

Like any community based epidemiologic investigation, there are numerous limitations inherent in the study design. Perhaps the most lingering question about this study is whether the quota sampling methodology may have also increased the between-country differences due to its reliance on proactive recruitment instead of using a preexisting sampling frame. Nonprobability samples have been shown to yield prevalence estimates for illicit drug use that are higher than probability surveys [48]. Therefore, this design used a diverse range of recruitment methodologies (e.g. street intercept, recruitment flyers, print advertisements), including proactive recruitment across diverse ranges of population strata. This design yields less bias in terms of coverage, nonresponse, and measurement than an internet panel study, but in theory introduces more bias than a probability sample. However, probability studies also face challenges because cellular telephones, privacy concerns, and identity threats significantly hamper their ability to recruit subjects [49]. This study conducted numerous validation checks by comparing our results with other known population-based studies of drug abuse in selected EU countries. The data were comparable, increasing confidence in study findings. For example, we subset our data to prevalence data obtained from reports sourced by the European Monitoring Centre for Drugs and Drug Addiction (EMCDDA). The data were sourced for persons of a similar age range in the same countries [50]. For cannabis, the EU estimate was 43.6 and the EU-Meds survey was 44.5 %, a difference of −0.9 percentage points. We also compared data from individual countries. To illustrate, the differences in amphetamine use, which includes prescription and illicit (e.g., methamphetamine) ranged from −2.8 percentage points to 2.2 percentage points. To illustrate, the estimate for Denmark, ages 15–34 in the 2014 EU Meds study was 7.5 %, but was 10.3 % for a study conducted in Denmark in 2011, reported by the EMCDDA. The survey methods were kept as comparable as possible across the countries, although it is possible that even subtle differences in the survey procedures (e.g. instrument translation, recruitment methods) may have increased the observed differences in the between-country estimates.

## Conclusions

With these limitations in mind, projects such as the current study can provide important comparative data for countries across the EU, the US, and beyond. The US is fortunate in that it has numerous systems and funding for drug abuse research that can be used to study substance use across geographic space and time. The EU drug abuse surveillance systems tend to focus less on human population-based studies, thereby making a direct comparison between the population rates in the US and the EU quite challenging. This study used the same base interview schedule that is also used by NSDUH, but modifications were made to accommodate cultural differences in terminology and self-administration. Thus, there are important methodological differences between the two studies that preclude direct comparisons. However, identification of the initial scope and prevalence of NMPDU in the EU is an important first step in building a worldwide system that can be used to monitor trends in substance use, track prominent risk and protective factors, and trace the transmission of information and products across national borders.

## Abbreviations

CI, confidence interval; EMCDDA, European Monitoring Center for Drug and Drug Abuse; EU, European Union; NMPDU, nonmedical prescription drug use; NSDUH, National Survey on Drug Use and Health; OR, odds ratio; RTI, Research Triangle Institute; UK, United Kingdom; US/USA, United States of America
